# Healthcare professionals’ perspective on managing the healthcare system in Croatia: a cross-sectional study

**DOI:** 10.3325/cmj.2024.65.339

**Published:** 2024-08

**Authors:** Stjepan Orešković, Vanesa Benković

**Affiliations:** 1 University of Zagreb School of Medicine, Andrija Štampar School of Public Health, Zagreb, Croatia; 2 Novo Nordisk Hrvatska, Zagreb, Croatia

## Abstract

**Aim:**

To determine Croatian healthcare (HC) professionals’ perspectives on HC management and leadership challenges.

**Methods:**

This cross-sectional study, conducted between May and June 2021, enrolled 1179 respondents from both the public and private sector, including medical doctors, nurses, pharmacists, economists, and other HC professionals. Their perspective on various facets of HC management, namely governance, ownership, accountability, financing, and potential for improvement, were investigated using an anonymous online survey.

**Results:**

Most respondents agreed that the system may be allocating resources ineffectively and that political mandates unduly influenced management decisions, impeding accountability. Competencies in organizational and financial experience, along with communication and leadership skills, were deemed fundamental for healthcare managers. Participants overwhelmingly supported data-driven decision-making, improved education, and the development of leadership skills as key avenues for system enhancement.

**Conclusions:**

The study underscores the need for better financial management, and overall governance, in the Croatian HC, offering insights that can inform evidence-based policy decisions and reforms toward a more efficient and accountable HC system.

Healthcare management is a profession that leads and organizes the delivery of healthcare (HC) services, making informed decisions to improve the health and well-being of individuals. Besides great demands and responsibility, this line of work also provides many rewards and personal satisfaction due to the ability to make a difference for other people (1). As Thompson et al put it, an overview of any HC management definitions should always include the major functions, roles, responsibilities, and competencies of HC managers (1,2).

Management of HC differs from other lines of work and other economic activities not only in the way that HC differs from other macroeconomic fields of expertise, but also due to its many complexities that require the coordination of many different specialties to enable seamless provision of healthcare.

The dimensions of HC management work may be observed from an external or internal focus (3). External focus is more oriented toward governance and ownership – usually related to stakeholder demands, private/public ownership, regulation, and public needs. Internally, the management is oriented toward staffing, budgeting, financial performance, patient satisfaction, provision of quality services, etc.

There is a vast body of research on how citizens perceive HC, the relationships with patients, financial and clinical organization of HC research, satisfaction with the system, and similar issues (4-6). However, in the literature we rarely find opinions by those who lead and comprise the core of the HC system and who have the highest impact in making any change work (7,8). These are not only physicians but also all other professionals working within the HC system.

Therefore, the aim of this study was to examine the opinions of the employees of the HC system in Croatia, both public and private, on the key challenges and quality of management and leadership, as well as on potential solutions for inadequate aspects. For this purpose, we investigated the following areas of HC system perception: key leadership/governance challenges in ownership and accountability of management personnel, HC financing challenges, areas for efficiency improvement, and financial risk protection.

## Materials and methods

A quantitative cross-sectional study was carried out. The questionnaire (Supplemental Material) was based on secondary data and related studies. This included the World Health Organization (WHO) framework of health system building blocks and insights from studies such as that by Thompson et al (1,9), which discuss the roles and competencies necessary for effective healthcare management. The questionnaire was pretested on a sample of 30 participants of various professions (doctors, nurses, administrators) in order to get a mix of perspectives. After completing the questionnaire, the respondents provided feedback on how they interpreted the questions and whether anything was confusing or unclear. The idea was that each question was easily understandable at first glance, and that everyone interpreted the question in the same way.

After that, we made some changes to ensure the questions were interpreted correctly. For example, we refined some of the wording and scales to be clearer and with less jargon. We also changed the order of some questions that seemed to flow better in a different sequence. After these changes, we ran a second, smaller round of pilot testing on a sample of different respondents (N = 8) to ensure our changes were effective.

The questionnaire consisted of five sections, with 30 questions in total. The first section included questions on demographic and professional characteristics, including sex, the year of birth, educational level, the size of the settlement where the respondent lived, profession, employment type, and duration of employment within the HC.

The section on HC system perception included a general question on the perception of its quality and management and a question on the comparison of the Croatian HC system with those of other EU countries.

The leadership/governance section included questions on the key issues, capacity to assemble and manage resources, required competencies of a HC manager, managerial knowledge and skills, ownership and accountability, and political dependency. The section on HC financing challenges included questions on an efficient and effective healthcare financing system, linkage of financial mobilization with an evidence-based plan, effective budget consumption, required financial resources to ensure sustainability, and interventions aimed at reducing wastage and enhancing cost-effectiveness. The section regarding the areas of improvement included questions on improved efficiency, financial risk protection, and decision-making.

The questionnaire parts that related to leadership, governance, and healthcare workforce were based on the WHO structure of health systems, which describes health systems in terms of six building blocks (service delivery, health workforce, information, medical products, vaccines and technologies, financing, and leadership/governance) (10). All questions had a predetermined list of answers, with an additional option for an open-ended response. These open-ended questions were assessed using combined deductive and inductive approaches. First, we set up some categories based on our research questions and the themes we expected to see (reflecting aspects of the WHO health system building blocks). This helped us quickly organize the responses. After this, we performed an inductive thematic analysis to capture emergent themes, making sure not to miss potential nuances.

Data were collected between May and June 2021 using www.1ka.si online platform. The target group consisted of different HC professionals and other HC employees, which included medical doctors (14 394 registered doctors in the Medical Atlas publication) (11), nurses (24 262 registered nurses according to Mihajlović et al) (12), pharmacists, economists, legal experts, psychologists, sociologists, data analysts, laboratory professionals, etc. Potential respondents were recruited via a third party (Agentius Ltd market research agency) in order to secure the participants’ anonymity. Participants had previously consented to be contacted for research purposes. This ensured the confidentiality and voluntary participation of all respondents. They received an e-mail with a link to the online survey, the introductory section of which served as informed consent form explaining the participants that had the right to withdraw from the study at any time. They were contacted via e-mails, and the Agency obtained the list from the official physician and nurse professional chambers, as well as through online professional communities (Facebook, LinkedIn, the homepage of the Leadership and Management of Healthcare Service Postgraduate Study at the University of Zagreb School of Medicine).

The final non-probabilistic, convenience sample of respondents answered the 30-question online questionnaire constructed for the purpose of this study. The inclusion criterion was employment in the healthcare system. Also, the study included medical doctors and pharmacists working in the pharmaceutical industry due to their close professional relationship with HC delivery and appropriate background education. Out of all the surveyed respondents (N = 3282), 1179 filled in the complete questionnaire (35.9%). 

Descriptive statistical procedures (categorical variables were presented as absolute numbers and percentages) were performed with SPSS, version 20 (IBM Corp., Armonk, NY, USA).

## Results

### Socio-demographic profile

A total of 1179 respondents were included in the study. The majority were women (842, 71%) and respondents in the age range from 29 to 61 years (n = 931, 79%), with smaller proportions in the age groups of ≥65 years (n = 100, 8.5%) and <29 years (n = 86, 7.3%; Table 1). The overall age range was from 20 to 81 years (mean 47 years; SD = 12.5). The largest proportion of participants were medical doctors (n = 657, 55.8%), followed by nurses and technicians (n = 224, 19%), pharmacists (n = 125, 10.6%), and economists (n = 65, 5.5%). The largest proportion of participants had a university degree (n = 583, 49.5%). A large number of participants worked as HC professionals between 16 and 35 years (n = 598, 50.7%; Table 1).

**Table 1:** Selected sociodemographic data of the sample

Variable	n	%
**Place of work**		
clinical hospital	250	21.2
general hospital	220	18.7
primary healthcare	310	26.3
special hospital	45	3.8
health insurance fund	12	1.0
Ministry of Health	2	0.2
private practice	68	5.8
pharmaceutical industry	123	10.4
other	149	12.6
total	1179	100.0
**Profession**		
doctor	657	55.8
nurse, technician	224	19.0
pharmacist	125	10.6
economist	65	5.5
psychologist	1	0.1
legal	18	1.5
sociologist	6	0.5
other	83	7.0
total	1179	100.0
**Education**		
high school	42	3.6
baccalaureate – 3 years	89	7.5
professional study	50	4.2
college	583	49.5
postgraduate study	261	22.1
doctorate	154	13.1
total	1179	100.0
**Years of employment**		
≤5	136	11.5
6-10	130	11.0
11-15	137	11.6
16-25	302	25.7
26-35	295	25.0
≥35	179	15.2
total	1179	100.0

### Perception of the HC system in general

The majority of respondents stated that the HC system was poorly led and organized, spending too much and delivering too little (56.1%). Overall, 33.8% of respondents reported that HC management was no better or worse than management in other sectors, while only 2.3% respondents viewed HC as a high-quality system serving patients’ needs well (Table 2). In the open-ended section relating to this question, average responses read along the lines of “excellent doctors, but poor management;” “led by politicians, not managers;” “good system, but poor leadership;” and “good medicine, bad management.”

**Table 2:** Perception of the healthcare (HC) system in Croatia by HC professionals

	N	%
**Croatian HC system is:**		
A high-quality HC system with good management that serves patient needs	27	2.3
Having the same management quality as other segments of society	399	33.8
Poorly led and organized system that spends too much and delivers too little	662	56.1
Something else	91	7.7
**Compared with other 35 HC systems in Europe:**		
Among the 5 best HC systems in Europe	12	1.0
Among the 10 best HC systems in Europe	281	23.8
Among the 10 least efficient HC systems in Europe	886	75.1

### Key leadership/governance challenges and competencies

Overall, 28.45% respondents identified poor management capacities of the Ministry of Health as the primary cause of problems in HC, and 23.6% identified the lack of educated staff in HC. With regard to whether HC problems were objectively large and hard to solve, participants were split in half, which indicates the complexity of healthcare management.

As many as 87.4% of respondents stated they had no influence on the selection of the head/director of the institution in which they worked (Table 3). As the key healthcare issue, 45.2% respondents identified the overdependence of the management structure on political mandates (45.2%), as well as the lack of accountability and responsibility for success and failure (25.5%). Other issues included a lack of management skills and knowledge among current managers (17.0%) and a lack of an incentive scheme (3.4%), whereas many open-ended answers stated that all four were equally important (Table 3). Overall, the most common answers to the open-ended questions read along the lines of freeing HC management from politics, which indicates a severe burden of politically-oriented leadership. Interestingly, a lack of courage was also listed in many open-ended claims.

**Table 3:** Perception of ownership, accountability in leadership, and governance of healthcare (HC) management

	N	%
**Your influence on the selection of the head/director of institution you work at is**		
major	41	3.5
minor	41	3.5
medium	66	5.6
none	1031	87.4
**The key HC system management issue is**		
lack of management skills and knowledge among current managers	200	17.0
lack of an incentive scheme	40	3.4
lack of accountability and responsibility for success and failures	301	25.5
overly large dependence of the management structure on political mandates	533	45.2
something else	104	8.8
**A good HC manager should possess the following knowledge:**		
knowing the cause and progression of diseases	197	16.7
knowing management theory and practice	434	36.8
organizational and financial experience	874	74.1
communication and leadership skills, regardless of the profession itself	469	39.8
something else	89	7.5

When asked which types of knowledge a healthcare manager should possess, out of four proposed answers, the majority selected organizational and financial experience (74.1%), followed by communication and leadership skills, regardless of the profession itself (39.8%; Table 3). In the open-ended answers, the respondents repeatedly emphasized having a vision, common-sense logic, ethics, political independence, and being uncorrupted.

When asked about the type of degree that people who run hospitals and clinics should have, most of the respondents mentioned postgraduate study in management, regardless of whether the primary education was in medicine (43%) or in economics (35%). The respondents who provided open-ended answers also stated that management experience was more valuable than education, and that personal values such as courage, ethics, morality, and being uncorrupt were more important than management skills. In the follow-up to this question, when asked which education a Minister of Health should possess, most of the respondents selected a medical degree with a master’s degree in business administration or a management diploma (54%).

### Healthcare financing challenges

As many as 66.2% of the respondents graded the management of HC resources as bad and as the area where key financial issues arose. A smaller number stated that the source of key financial issues was resource scarcity in HC (14.6%), the state not fulfilling its (financial) obligations toward a major health insurer (4.7%), or too much money being spent on pharmaceuticals (2.5%; Table 4).

**Table 4:** Perceived financing challenges in the healthcare system

	N	%
Key financial issues arise from:		
too few resources invested in healthcare	172	14.6
state not fulfilling its obligations toward the Health Insurance Fund	55	4.7
too much money spent on drugs	29	2.5
too much money spent on hospital care	15	1.3
bad management of existing resources	780	66.2
something else	128	10.9
Which financial measure would improve healthcare system quality?		
increase taxes and reroute them to healthcare	58	4.9
increase contributions from salaries (which are now 16.5%)	44	3.7
increase co-payments for drugs and services	222	18.8
offer part of public services to private market	427	36.2
expenditure is irrational in any case and management quality is at fault for that	765	64.9
something else	126	10.7
Healthcare managers should		
receive a regular salary like all other healthcare workers	392	33.2
receive the highest salary in a given institution	70	5.9
receive the highest salary and special financial bonuses as performance incentives	260	22.1
receive rewards such as additional education as performance incentives	343	29.1
something else	113	9.6

When they needed to select a financial measure that would improve the quality of the HC system, the majority of HC professionals agreed that healthcare expenditure was irrational in any case, and that management quality was at fault for that (64.9%). Overall, HC professionals stated that the solution was to offer part of public services to the private market (36.2%), increasing co-payments for drugs and services (18.8%), increasing taxes and rerouting them to healthcare (4.9%), while 3.7% stated that the solution was to increase healthcare contributions from salaries (which are now at 16.5%) (Table 4).

In the section on salary incentives and rewards, respondents showed resistance toward giving an extra bonus to managers. The majority stated that managers should receive salaries like all other healthcare workers (33.2%) or receive additional education as performance incentives (29.1%). Some of the respondents opted for special financial bonuses as performance incentives (22.1%) or for the managers to receive the highest salary in a given institution (5.9%; Table 4). In the open-ended section (9.6%), there were many suggestions for bonuses for proven improved efficiency, for mitigating corruption with a big enough salary, or for accountability for bad management, not just bonuses for success.

### Areas of improvement

The majority of the respondents answered that using more data and information in decision-making would result in improvements (93.3%), similar to better education and leadership, and management skills (94.1%; Table 5). The majority of respondents (89.2%) repeatedly selected better management autonomy from political structures. In the quite long list of open-ended answers provided (24.3%), the following ideas were identified: pay per performance and monitoring outcomes of treatment, transparent employment policies, better definitions of private and public engagement, increasing the autonomy of nurses, less politics, awareness of the patient’s journey, decreasing corruption in tendering, increasing the number of healthcare insurance companies, better organization, better data and digital data usage, and more responsibility placed on the patients.

**Table 5:** Areas of improvement of the healthcare system managing in Croatia

Areas of improvement	Agree	Disagree
more financial income	675 (57.3)	504 (42.7)
more employed doctors and nurses	924 (78.4)	255 (21.6)
more employment of other healthcare workers	623 (52.9)	556 (47.2)
better education and management, and leadership skills	1109 (94.1)	70 (5.9)
better management autonomy from political structures	1052 (89.2)	127 (10.8)
more use of data and information in decision-making	1101 (93.3)	78 (6.6)
something else	286 (24.3)	84 (7.1)
**Healthcare management would have more efficient management if:**		
the Ministry of Health had more jurisdiction in decision-making	380 (32.3)	799 (67.8)
the Croatian Health Insurance Fund had more jurisdiction in decision-making	190 (16.1)	989 (83.9)
hospital heads had more jurisdiction in decision-making	531 (45.1)	648 (55.0)
There is enough of jurisdiction and power for decision-making, but these are inadequately used and implemented.	929 (78.8)	250 (21.2)
something else	224 (19.0)	96 (8.1)
**Key decision-making in healthcare system should be based on:**		
using data analysis of disease trends and hospital performance	1119 (94.9)	60 (5.1)
autonomous decisions of hospital heads	405 (34.6)	774 (65.6)
complying to political leadership that selected hospital heads	24 (2.1)	1155 (97.7)
complying to political leadership and economic lobbies	27 (2.3)	1152 (97.7)
team decision-making	1118 (94.9)	61 (5.2)
something else	181 (15.4)	104 (8.8)

The majority of respondents identified the use of data analysis of disease trends and hospital performance as the key tool in decision-making (94.9%; Table 4). Respondents unanimously emphasized the importance of not complying with political leadership, and economic lobbies (97.7%), and the need for team decision-making (94.9%).

## Discussion

HC professionals included in this study believed that the management of the existing resources was poor and the current HC system in Croatia was inefficient and plagued by political interference in management decisions. The majority of our respondents worked in the HC system for 16-35 years. Hence, they are well familiar with the system.

To further understand our respondents’ perspective, one must take into account that the Croatian HC system derives from a unique societal experiment of socialism, with the foundation laid by Andrija Štampar, co-founder of the WHO, which made Croatia and Slovenia the most advanced socialist economies. Consequently, the dominant national political narrative is that Croatian HC system is among the best in the world. This partly explains why Croatia is actually globally ranked as a top country for transplantation (13), and this is seen as a quality applicable throughout the whole system.

The key leadership challenges in ownership and accountability of healthcare management identified in this study point toward what Taleb describes as “The curse of modernity is that we are increasingly populated by a class of people who are better at explaining than understanding, or better at explaining than doing” (14). Thus, responsibility and accountability are often emphasized, but not always implemented. Furthermore, participants reported a perceived lack of accountability and responsibility for success and failures. This finding emphasizes once again that in highly bureaucratic systems where there is performance-related measurement, the person is separated from the consequences of his or her actions, truly having no skin in the game.

The financing of HC systems was an ongoing theme in many reform efforts not only in Croatia, but also in central and eastern Europe. Many countries distanced themselves from the centralized state model and shifted toward decentralized and contracted social health insurance, which in Croatia is a mix of the Bismarck and Beveridge models, financed predominantly from contributions, and less from taxes.

A majority of respondents in this study perceived bad management of healthcare resources as the source of key financial issues. When the respondents were asked to identify a measure that would improve healthcare system quality, most of them agreed that healthcare expenditure was irrational in any case, and that management quality was at fault. A similar view has been expressed by a former Minister of Finance (2015-2022), to paraphrase: a large amount of money is invested in repairing health debts, however healthcare sinks into even deeper expenditure debt (15). Our respondents saw the solution in offering part of public services to the private market (36.2%), increasing co-payments for drugs and services (18.8%), increasing taxes and rerouting them to healthcare (4.9%), while a minority (3.7%) believed that the solution was to increase contributions from salaries (which are now 16.5%). The current Minister of Health proposed increasing healthcare contributions, although Croatia already is among countries with the highest contributions, higher than Germany (16). Despite HC employees’ belief that the Croatian healthcare system abounds with all resources except good management, the Health Minister still sees this as a spending issue, and is creating further pressure on the economy and working citizens. Employees of the HC system seem to be aware of what the Minister of Finance showed through data – that lack of financing is not the issue, but a lack of resource management.

According to research from 2011-2018, despite a persistent recession, the private healthcare sector in Croatia experienced an average annual growth rate of 10% (17). Almost all EU countries, not only Croatia, are facing healthcare cost control and quality control challenges. Croatia’s National Healthcare Strategy 2012-20 aimed to improve the efficiency and effectiveness of the health system, but according to European Commission, few of these measures have been implemented (18).

In the context of evaluating the major findings of the study, Buljan and Simovic's (19) study provides an important benchmark. Their research, which examined the overall efficiency of healthcare expenditures in relation to average life expectancy in Croatia and 21 other EU countries, reveals a significant efficiency gap. Specifically, in 2018, Croatia's efficiency was a mere 57%, significantly lower than the EU average of 88%. While the Croatian HC system achieved a cost-effectiveness rate of 100%, its systemic effectiveness was notably lower, at just 48% (19). This disparity underscores a broader theme identified in our study – while Croatia allocates substantial resources to healthcare, the conversion of these resources into effective health outcomes is markedly inefficient. This conclusion aligns with the perceptions of HC professionals in our survey, who criticized the management of resources and echoed a negative impact of political mandates on healthcare efficiency. Buljan and Simovic's findings reinforce the insights from our data, suggesting that the Croatian HC system could achieve similar health outcomes with significantly less resource expenditure and pointing to deeply rooted inefficiencies in resource management. So, the key question one might ask is how to convert available material and human resources into an efficient HC system.

Participants strongly supported data-driven decision-making and improved education as key directions for system enhancement: using more data and information in decision-making would result in improvement. This finding is not a surprise given that the continuous transition to digital health promises to improve and transform healthcare (20). Using machine learning to analyze health data enhances clinician-patient interactions, enabling quicker and more evidence-informed decisions. It also improves operational efficiencies, patient outcomes, and the cost-effectiveness of care delivery (21). The emphasis on education and training in data analysis also indicates that HC professionals should be taught the necessary skills and knowledge to interpret and apply data analytics effectively (22). A high proportion of answers indicated the need for education on leadership and management skills (94.1%). Healthcare management education should be obligatory for all personnel holding healthcare management positions, whether in the hospital or administrative ones. Organizational culture derives from both defensive mechanisms (such as resistance to change) and managerial actions (23,24). This might partially explain the resistance to change in hospital and general healthcare management. These findings are in line with the literature emphasizing the need for effective leadership and strategic (and transformational) management in the healthcare setting (23).

In studies on strategic capabilities, communication, integrity, and accountability were also perceived as important leadership and interpersonal skills needed to effectively lead healthcare teams and drive organizational change (23). Mintzberg describes how effective management is as much about people as it is about operations, particularly in healthcare settings where collaboration is crucial (25). He argues that the real challenge for healthcare managers is to balance professional competence with interpersonal empathy, ensuring that operational efficiency does not fully shadow patient-centric care. We might say that all of the above relates to good communication. In a similar way, transformative leadership (inspiring and motivating teams) is highly effective in HC environments. Kouzes and Posner discuss how leadership is about mobilizing others to fight for shared goals; translated to HC this means working collaboratively to improve patient outcomes and system efficiency (26). These perspectives suggest that HC organizations should prioritize the development of management training programs that focus on both the strategic and human elements of leadership.

At the same time, efficient managerial actions should require higher management autonomy from politics. Respondents also agreed that better management autonomy from political structures was important. This dimension may be better understood as related to (political) trust. Political distrust has been the norm, rather than the exception, in many established democracies in recent decades (27), and is intertwined with the failure of governance, particularly in the HC system. The relationship between performance evaluations and distrust is reciprocal (28,29). In this sense, HC administrations should aim for better educated governance and communication skills, which might improve political trust.

Several limitations of the study must be acknowledged. First, the survey's reliance on self-reported data may introduce a degree of bias, as participants might have differing interpretations of the questions or might respond in ways they perceive as socially acceptable rather than completely truthful. Although authors made efforts to mitigate this through question design and anonymity, such biases cannot be entirely eliminated. Second, the study's cross-sectional design limits the ability to draw causal inferences from the data. These provide a snapshot of opinions and perceptions at a specific point in time, but they do not indicate trends or changes over time. Future studies might take a longitudinal approach to track changes in perceptions as reforms and interventions are implemented. Third, although the sample was intended to be representative, the non-random nature of the participant recruitment did impact the generalizability of the results. Survey participants were recruited through professional networks and associations, which may not fully represent the broader population of HC professionals in Croatia.

In response to these limitations, future studies should consider employing a mixed-methods approach, combining quantitative surveys with in-depth interviews or focus groups. This approach could not only be used to validate our observations but also to provide a deeper understanding of the reasons behind them.

Healthcare institutions and the educational sector may profit from the opinions expressed by respondents in this study, and may also use their own approach based on the WHO building blocks structure. Using established WHO global health commitments might steer sustainability and ownership in a more concrete and focused way than current political promises.

To the political leadership presenting a narrative of introducing changes and the need for professionalism and evidence, this study shows that employees are in fact allies of efforts to introduce changes to the HC system. Those employed in HC are often seen as resisting change, but their values and orientations are directed toward systems based on knowledge, decision-making, and inclusiveness.

In summary, participants' consensus on resource allocation challenges, driven by poor management and organizational inefficiencies, calls for effective strategies to optimize resource use. Additionally, concerns about political influences on HC management decisions signal a demand for greater accountability and evidence-based policies. Respondents recognized the importance of organizational and financial experience, along with communication and leadership skills, which emphasizes the value of educational and training programs for HC managers that focus on both strategic and human elements of leadership. The study strongly advocates for data-driven decision-making, encouraging the incorporation of data analysis and performance measurement in HC policy decisions. In conclusion, this study offers crucial insights for policymakers, researchers, and HC professionals. By addressing these issues, the Croatian HC system can move toward greater efficiency and accountability, ultimately better serving the health needs of its population.
